# Clonal Hematopoiesis does not influence manufacturing of Chimeric Antigen Receptor (CAR) T-Cells

**DOI:** 10.1038/s41409-026-02924-y

**Published:** 2026-06-03

**Authors:** Simon M. Krauß, Konstantin Weibl, Enrica Bach, Mandy Brückner, Anne Weigert, Theresa Tumewu, Elena Ruschpler, Gunhild Vogtmann, Sandra Hoffmann, Olaf Penack, Martin Janz, Raymund Buhmann, Reinhard Henschler, Lars Bullinger, Ulrich Keller, Sebastian Schwind, Maximilian Merz, Madlen Jentzsch, Georg-Nikolaus Franke, Marco Herling, Uwe Platzbecker, Klaus H. Metzeler, Vladan Vučinić

**Affiliations:** 1 Department of Hematology, Cell Therapy, Hemostaseology and Infectiology, University of Leipzig Medical Center, Leipzig, Germany; 2 Comprehensive Cancer Center Central Germany (CCCG), Leipzig, Germany; 3 Department of Hematology, Oncology and Cancer Immunology (Campus Virchow-Klinikum), Charité - Universitätsmedizin Berlin, corporate member of Freie Universität Berlin and Humboldt-Universität zu Berlin, Berlin, Germany; 4 Department of Hematology, Oncology and Cancer Immunology (Campus Benjamin Franklin), Charité - Universitätsmedizin Berlin, Berlin, Germany; 5 Institute for Transfusion Medicine, University of Leipzig, Leipzig Medical Center, Leipzig, Germany; 6 German Cancer Consortium (DKTK) partner site Berlin, German Cancer Research Center (DKFZ), Heidelberg, Germany; 7 National Center for Tumor Diseases (NCT) partner site Berlin, German Cancer Research Center (DKFZ), Heidelberg, Germany; 8 Max-Delbrück-Center for Molecular Medicine, Berlin, Germany; 9 Cluster of Excellence ImmunoPreCept, Charité - Universitätsmedizin Berlin, Berlin, Germany; 10 University Hospital Carl Gustav Carus, Technische Universität Dresden, Dresden, Germany; 11 Fraunhofer Institute for Cell Therapy and Immunology, Leipzig, Germany

**Keywords:** Translational research, B-cell lymphoma, Genetics research, Cancer immunotherapy

Treatment with chimeric antigen receptor (CAR) T-cells has proven as a highly effective option for treatment of patients with lymphoproliferative diseases and plasma cell dyscrasia. Tisagenlecleucel (tisa-cel; Novartis AG, Basel, Switzerland) is approved for treatment of patients with large B-cell lymphoma (LBCL), precursor acute lymphoblastic leukemia and follicular lymphoma after two previous treatment lines [1]. The manufacturing process of CAR T-cells and starts with leukapheresis of mononuclear cells, followed by ex vivo transduction of the CAR receptor, and subsequent expansion at the production site [2]. However, not all collections result in products manufactured according to specifications, with deviations resulting in out of specification (OOS) products or the termination of production. Our group recently reported about the influence of cellular composition and T-cell senescence on manufacturing success of CAR T-cells [3].

Factors like chronic replicative stress or exposure to DNA-damaging agents, like chemotherapy, lead to accumulation of somatic mutations. Some of these mutations confer a selective fitness advantage and consequently lead to the outgrowth of a clonal population, coined clonal hematopoiesis (CH) [4, 5]. We hypothesized that the presence of CH in a heavily pretreated patient population could affect the manufacturing process of CAR T-cells, and report here on a retrospective analysis of the influence of CH on the manufacturing success of tisa-cel in the previously reported cohort. To the best of our knowledge, this question has not yet been examined systematically. We used a custom, highly sensitive next generation sequencing assay based on single-molecule molecular inversion probes to measure CH mutations as described previously [6], to analyze mononuclear cell collections of 49 patients (37 male) scheduled to undergo treatment with tisa-cel, which were shipped for manufacturing between May 2019 and April 2022. All methods were performed in accordance with the relevant guidelines and regulations. Patients were median 61 years old (range, 20–80) and had received median 4 treatment lines (range, 2–7) prior to apheresis (Table S1). CH-related mutations in apheresis material were detected in 23/49 patients (47%), which is in line with previous reports about CH prevalence in populations treated with CAR T-cells [7, 8]. Median variant allele frequency (VAF) was 1.6% (mean, 5.7%), median number of mutations in patients with CH was 2 (range, 1–6) and CH prevalence increased with patient age, but did not associate with number of prior treatment lines, previous bendamustine treatment or bone marrow infiltration (Fig. S1). Presence of CH was neither associated with WBC, ALC or CD3^+^ cell count before apheresis nor with CD3^+^, CD3^+^CD4^+^ or CD3^+^CD8^+^ cell yields in collections.CH mutations with VAF < 2% were identified in 10 (20%), and mutations with VAF ≥ 2% in 13 collections (27%; Table S2). Overall, the most frequently mutated genes in our cohort were *DNMT3A* (12/49, 24%), *PPM1D* (11/49, 22%) and *TET2* (9/49, 18%).

Manufacturing of CAR T-cells was successful in 34 collections (70%; “successful collections”), with 7 terminations (14%) and 8 OOS products (16%; Table S3), which is higher than reported in the questionnaire of the European Bone Marrow Transplantation (EBMT) Registry [9]. CH prevalence was comparable between groups, with 17/34 (50%) successful collections, 4/7 (57%) of terminations and 2/8 of OOS (25%) harboring CH mutations. Similarly, number of mutations, mutation distribution across genes and median VAF did not differ between groups (Figs. 1 & S3). Further analysis of collections with mutations ≥ 2% VAF, to ensure comparability with prior analysis of clonal hematopoiesis of indeterminate potential (CHIP), yielded similar results (Figs. S4, S5).

**Fig. 1:**
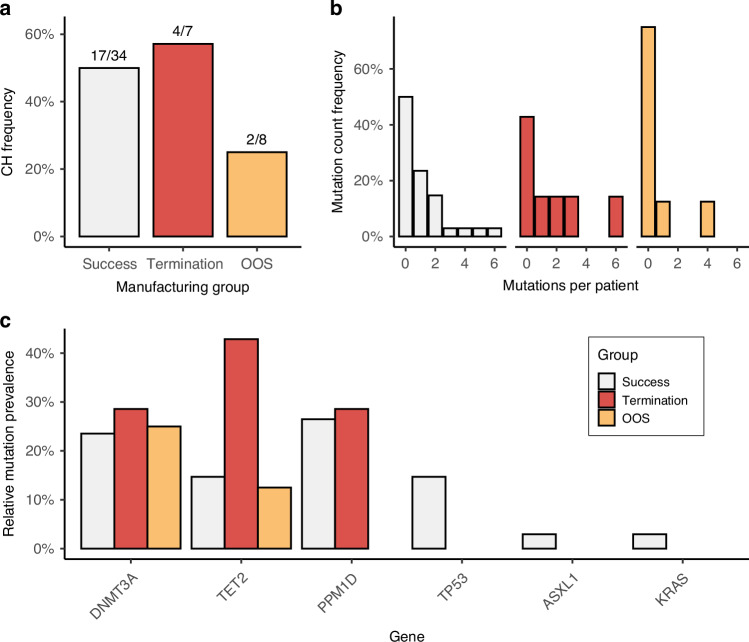
Presence of clonal hematopoiesis (CH) and association with manufacturing results. **a** Barplot comparing CH prevalence according to manufacturing results. **b** Histogram comparing mutation count per patient between manufacturing result groups. **c** Barplot comparing relative mutation prevalence across genes within each manufacturing group. OOS out-of-specification.

Also, when restricting the analysis to proliferation-related manufacturing failures, CH mutations were detected in 4/8 cases (50%) compared to 19/41 (46%) cases of successful production. No association between CH status and proliferation-related manufacturing failure was observed (*p* = 1).

The presence of CH has been previously evaluated in context of outcomes of patients undergoing CAR T-cell therapy. However, data regarding this issue are still relatively scarce and conflicting. In a retrospective unicentric analysis of 32 patients, Teipel et al. reported that the presence of CH was associated with better overall survival, but noticed no associations with the overall response or incidence of inflammatory complications [8]. Similarly, Miller et al. reported the presence of CH in 48% of 154 patients undergoing treatment with different CAR T-cell products for lymphoma and myeloma [10]. This analysis confirmed the association of CH with higher rates of complete remissions after treatment but also with higher severity of cytokine release syndrome in patients younger than 60 years. Saini et al. could not confirm these observations. In their cohort of 114 patients with large B-cell lymphoma treated with anti-CD19 CAR T-cells, the presence of CH had no influence on outcomes or on the overall incidence of CRS or ICANS [11]. Another German multicentric analysis of 110 patients with lymphoma and ALL undergoing treatment with anti-CD19 CAR T-cells measured a CH prevalence of 56.4%, but detected no difference in outcome or prevalence of either CRS or ICANS. In further follow-up, 100 days after CAR T-cell infusion a modest VAF increase of 1.3% and acquisition of novel mutations was detected [7].

We also analyzed the potential influence of therapy-related DNA damage response (DDR) mutations on manufacturing success. Overall, 13 patients (27%) carried mutations in the DDR genes *PPM1D* or *TP53*, of which 11 were in the group of successful production and 2 in the termination group. There was no significant difference in DDR status by manufacturing group, by number of prior treatment lines or by previous treatment with bendamustine (Fig. S6). Given the limited cohort size, the absence of association of DDR mutations and manufacturing of CAR T-cells does not exclude a modest biological effect.

OOS and termination events are relevant issues with consequences for the treatment of patients, but also have financial consequences as the reimbursement of the OOS products is often denied by payers [12]. As apheresis is the starting step for the production of CAR T-cells, there is an urgent need for systematic analyses of functional characteristics of collected cells and their influence on the production process.

In contrast to previously reported data regarding associations of CH with clinical outcomes, our results focus on the manufacturing process, and show no measurable influence of CH. Different from previous reports, which measured CH in peripheral blood or bone marrow aspirate, we analyzed mononuclear cells from cryopreserved leukapheresis material, which is the actual starting material for CAR T-cell production.

In addition to its retrospective nature, our analysis has several limitations. First, it focuses on a heavily pretreated population of patients after multiple lines of immune-chemotherapy and was restricted to one CAR T-product. However, as apheresis modalities are largely comparable across CAR T-products, our findings regarding CH in the starting material may be generalizable beyond tisa-cell. Secondly, the relatively small cohort, particularly in the termination and OOS subgroups, drastically limited statistical power, most findings are descriptive. Nonetheless, the absence of any measurable trend across several analytical layers suggests that CH-related effects, if present, are likely minor under current manufacturing conditions.

CH is frequent among patients referred for CAR T-cell therapy, but does not appear to compromise the manufacturing process of tisa-cel. These results do not support the need for CH screening during manufacturing feasibility assessments and highlight that most production failures are probably driven by (pre)-analytical or technical factors rather than patient-related CH. Future studies integrating CH profiling in combination with immunophenotyping and process analytics will be instrumental in validating these findings.

## Supplementary information

Supplemental Material (fused with tables and Figures)

## Data Availability

The datasets generated during and/or analyzed during the current study are available from the corresponding author on reasonable request due to privacy/ethical reasons.
