# Supplementary material for: Clonal Hematopoiesis does not influence manufacturing of Chimeric Antigen Receptor (CAR) T-Cells
Source: Bone Marrow Transplant. 2026 Jun 3;61(8):1105–7. doi: 10.1038/s41409-026-02924-y (PMC13437239; doi:10.1038/s41409-026-02924-y)
Supplement: Supplementary file 1 [file 41409_2026_2924_MOESM1_ESM.docx]

Supplemental appendix

Clonal Hematopoiesis does not influence manufacturing of
Chimeric Antigen Receptor (CAR) T-cells

# Supplemental Methods

## Apheresis

All patients underwent an assessment of complete blood counts, including white blood cell count (WBC), absolute lymphocyte count (ALC) and circulating CD3+ cell count, prior to apheresis. All collections were performed with the Spectra Optia Apheresis system (Terumo BCT, Tokyo, Japan) using the continuous MNC program (version 11). The apheresis volume was set at three-times of the estimated blood volume, and collection time was limited to maximum five hours according to national standards.^1^ The collections were performed via peripheral venous catheters. Acid-citrate-dextrose was used as anticoagulant at inlet/outlet ratio of 12:1 - 15:1. Serum calcium levels were assessed routinely during apheresis, and calcium substituted as clinically indicated. All collections were stored in cryopreservation media with 5% dimethyl sulfoxide according to previously published data and preserved in the vapor phase of liquid nitrogen.^2,3^ Concentrations of CD3+, CD4+ and CD8+ cells in mononuclear cell collections were determined by flow cytometry on the day of apheresis as described previously.^4^

## Analysis of Clonal Hematopoesis

Genetic variants associated with CH were identified from genomic DNA extracted from the leukapheresis collections. Next generation sequencing (NGS) was performed via a custom single-molecule Molecular Inversion Probe (smMIP) assay covering 82 regions in 24 known CH and acute myeloid leukemia (AML)-related genes as described previously.^5^ The assay reached a median sequencing depth of 7860x before and 550x after UMI-deduplication, with 95% of the target regions being covered ≥690x before and ≥60x after UMI-deduplication. The sensitivity was 0.5% VAF.

## Statistics

Clinical and molecular data were used to analyze associations between CH status and manufacturing results of CAR T-cells. Associations with continuous parameters were compared using the Kruskal–Wallis or Wilcoxon rank-sum tests, and those with categorical variables using Fisher’s exact test. Associations of CH status with age was modelled using univariable logistic regression. Differences between groups were considered significant at p-values ≤ 0.05. When appropriate, p-values were adjusted to control the false discovery rate (FDR) using the Benjamini-Hochberg method, and adjusted p-values ≤ 0.05 were considered statistically significant. All statistical analyses were performed using R (version 4.4.1).^6^ Color palettes for figures were optimized using color vision deficiency simulations to ensure distinguishability for protanopia, deuteranopia, tritanopia and deuteranomaly.

# Supplemental Results

## Manufacturing failures - out of specification (OOS) events and terminations

The manufacturing failures consisted of 8 OOS productions, either due to indeterminate mycoplasma testing (n = 6) or insufficient proliferation (n = 2), and 7 production terminations, due to microbiological contamination (n = 1) and insufficient proliferation or expansion (n = 6).

## Cellular assessment of peripheral blood and apheresis

The median WBC in peripheral blood was 5.6 (range, 1.0 - 31.5) x10^9^/L, with a median ALC of 0.7 (range, 0.1 - 5.5) x10^9^/L and a median CD3^+^ cell count of 0.7 (range, 0.1 - 5.2) x10^9^/L. There was no difference in blood counts by manufacturing groups (Table S3).

In mononuclear blood cell collections, median collected CD3^+^ yield was 4.9 (range, 1.0 - 32.0) x10^9^ cells and was significantly lower in the termination (adjusted p = 0.036) and OOS (adjusted p = 0.036) groups compared to the success group. Median CD3^+^CD4^+^ yield was 2.3 (range, 0.3 - 10.3) x10^9^ and median CD3^+^CD8^+^ yield was 2.7 (range, 0.4 - 26.7) x10^9^ and did not differ between manufacturing groups (Figure S7; Table S3).

# Supplemental References

**1	Beschluss der Bundesärztekammer über die erste Fortschreibung der Richtlinie zur Herstellung und Anwendung von hämatopoetischen Stammzellzubereitungen. *Deutsches Ärzteblatt Online* 2019.**

**2	Berz D, McCormack EM, Winer ES, Colvin GA, Quesenberry PJ. Cryopreservation of hematopoietic stem cells. *Am J Hematol* 2007; 82: 463–72.**

**3	Leemhuis T, Padley D, Keever-Taylor C, Niederwieser D, Teshima T, Lanza F *et al.* Essential requirements for setting up a stem cell processing laboratory. *Bone Marrow Transplant* 2014; 49: 1098–105.**

**4	Koehl U, Bochennek K, Esser R, Brinkmann A, Quaritsch R, Becker M *et al.* ISHAGE-based single-platform flowcytometric analysis for measurement of absolute viable T cells in fresh or cryopreserved products: CD34/CD133 selected or CD3/CD19 depleted stem cells, DLI and purified CD56+CD3− NK cells. *Int J Hematol* 2008; 87: 98–105.**

**5	Krauß SM, Telzerow E, Richter D, Moret AS, Rothenberg-Thurley M, Sauerland C *et al.* Clonal hematopoiesis in AML long-term survivors: Risk factors and clinical consequences. *Hemasphere* 2025; 9: e70183.**

**6	R Core Team (2020). R: A language and environment for statistical computing. R Foundation for Statistical Computing, Vienna, Austria. https://www.r-project.org/.**

# Supplemental tables

## Table S1: Patient characteristics and manufacturing results

| **Variable** | **Overall** | **no CH** | **CH** | **p** |
| --- | --- | --- | --- | --- |
| **Patient number** | N = 49^1^ | n = 26^1^ | n = 23^1^ |  |
| **Age at apharesis [y]** | 61 (20, 80) | 57 (30, 80) | 62 (20, 79) | 0.078² |
| **Sex** |  |  |  | >0.9³ |
| *female* | 12 (100%) | 6 (50%) | 6 (50%) |  |
| *male* | 37 (100%) | 20 (54%) | 17 (46%) |  |
| **Diagnosis** |  |  |  | 0.5³ |
| *LBCL* | 48 | 26 | 22 |  |
| *ALL* | 1 | 0 | 1 |  |
| **Prior therapy lines** | 4.00 (2.00, 7.00) | 4.00 (2.00, 6.00) | 3.00 (2.00, 7.00) | 0.7² |
| **Bendamustine treatment** | 15 (100%) | 7 (47%) | 8 (53%) | 0.8³ |
| **Bone marrow infiltration** | 9 (100%) | 6 (67%) | 3 (33%) | 0.5³ |
| **Manufacturing results** |  |  |  | 0.4³ |
| *success* | 34 (100%) | 17 (50%) | 17 (50%) |  |
| *termination* | 7 (100%) | 3 (43%) | 4 (57%) |  |
| *OOS* | 8 (100%) | 6 (75%) | 2 (25%) |  |

^1^ Median (range); n (row %); n

² Wilcoxon rank sum test

³ Fisher’s exact test

Abbreviations: CH, clonal hematopoiesis; LBCL, large B-cell lymphoma; ALL, precursor B-cell acute lymphoblastic leukemia; OOS, out-of-specification

## Table S2: CH ≥ 2% VAF: patient characteristics and manufacturing results

| **Variable** | **Overall** | **no CH ≥ 2%** | **CH ≥ 2%** | **p** |
| --- | --- | --- | --- | --- |
| **Patient number** | N = 49^1^ | n = 36^1^ | n = 13^1^ |  |
| **Age at apheresis [y]** | 61 (20, 80) | 60 (30, 80) | 67 (20, 78) | 0.2² |
| **Sex** |  |  |  | 0.7³ |
| female | 12 (100%) | 8 (67%) | 4 (33%) |  |
| male | 37 (100%) | 28 (76%) | 9 (24%) |  |
| **Diagnosis** |  |  |  | 0.3³ |
| LBCL | 48 | 36 | 12 |  |
| ALL | 1 | 0 | 1 |  |
| **Prior therapy lines** | 4.00 (2.00, 7.00) | 4.00 (2.00, 7.00) | 3.00 (2.00, 5.00) | 0.13² |
| **Bendamustine treatment** | 15 (100%) | 10 (67%) | 5 (33%) | 0.7³ |
| **Bone marrow infiltration** | 9 (100%) | 7 (78%) | 2 (22%) | >0.9³ |
| **Manufacturing status** |  |  |  | 0.5³ |
| success | 34 (100%) | 26 (76%) | 8 (24%) |  |
| termition | 7 (100%) | 4 (57%) | 3 (43%) |  |
| OOS | 8 (100%) | 6 (75%) | 2 (25%) |  |

^1^ Median (range); n (row %); n
^2^ Wilcoxon rank sum test
^3^ Fisher’s exact test

Abbreviations: CH, clonal hematopoiesis; LBCL, large B-cell lymphoma; ALL, precursor B-cell acute lymphoblastic leukemia; OOS, out-of-specification

## Table S3: Out of specification products and clinical factors

| **Variable** | **Overall** | **indeterminate mycoplasma** | **insufficient proliferation** | **p** |
| --- | --- | --- | --- | --- |
| **Patient number** | N = 8^1^ | n = 6^1^ | n = 2^1^ |  |
| **Age at apharesis [y]** | 55 (39, 77) | 64 (42, 77) | 40 (39, 40) | **0.071²** |
| **Sex** |  |  |  | >0.9³ |
| *female* | 4 (100%) | 3 (75%) | 1 (25%) |  |
| *male* | 4 (100%) | 3 (75%) | 1 (25%) |  |
| **Diagnosis** |  |  |  | >0.9³ |
| *LBCL* | 8 | 6 | 2 |  |
| *ALL* | 0 | 0 | 0 |  |
| **Prior therapy lines** | 3.00 (2.00, 4.00) | 3.00 (2.00, 4.00) | 4.00 (4.00, 4.00) | 0.11² |
| **Bendamustine treatment** | 3 (100%) | 1 (33%) | 2 (67%) | 0.11³ |
| **Bone marrow infiltration** | 3 (100%) | 2 (67%) | 1 (33%) | >0.9³ |
| **CH status** |  |  |  | >0.9³ |
| *no CH* | 6 (100%) | 4 (67%) | 2 (33%) |  |
| *CH <2%* | 0 (0%) | 0 (0%) | 0 (0%) |  |
| *CH >=2%* | 2 (100%) | 2 (100%) | 0 (0%) |  |

^1^ Median (range); n (row %); n
^2^ Wilcoxon rank sum test
^3^ Fisher’s exact test

Abbreviations: LBCL, large B-cell lymphoma; ALL, precursor B-cell acute lymphoblastic leukemia; OOS, out-of-specification; CH, clonal hematopoiesis

## Table S4: Bloodcounts and cell yields

| **Variable** | **Overall** | **success** | **termination** | **OOS** | **P**² |
| --- | --- | --- | --- | --- | --- |
| **Patient number** | N = 49^1^ | n = 34^1^ | n = 7^1^ | n = 8^1^ |  |
| **Peripheral blood counts (x10^9/L)** | |  |  |  |  |
| WBC | 5.6 (1.0, 31.5) | 6.0 (1.9, 31.5) | 5.2 (2.3, 10.7) | 4.6 (1.0, 7.5) | 0.2 |
| ALC | 0.7 (0.1, 5.5) | 0.7 (0.1, 5.5) | 0.6 (0.3, 1.1) | 0.5 (0.3, 2.5) | 0.13 |
| CD3+ cell count | 0.7 (0.1, 5.2) | 0.8 (0.1, 5.2) | 0.7 (0.3, 1.0) | 0.3 (0.2, 0.9) | 0.3 |
| **Collection cell yields (x10^9/L)** | |  |  |  |  |
| CD3+ yields | 4.9 (1.0, 32.0) | 6.0 (1.0, 32.0) | 4.0 (1.7, 6.0) | 2.7 (1.6, 18.5) | **0.013** |
| CD3+CD4+ yields | 2.3 (0.3, 10.3) | 2.6 (0.3, 10.3) | 1.6 (0.8, 2.7) | 2.0 (0.3, 4.3) | 0.2 |
| CD3+CD8+ yields | 2.7 (0.4, 26.7) | 3.4 (0.4, 26.7) | 2.5 (0.4, 3.4) | 1.1 (0.4, 5.5) | 0.061 |

^1^ Median (range)

^2^ Kruskall-Wallis rank sum test

Abbreviations: WBC, white blood cell count; ALC, absolute lymphocyte count

# Supplemental figures

## Figure S1

Overall CH prevalence in this cohort and associations with clinical factors. **(A)** Violin-boxplot showing VAF distribution of CH mutations. **(B)** Histogram showing distribution of mutation count per patient. **(C)** Barplot displaying CH prevalence according to patient age group. (**D, E, F**) Barplot displaying CH prevalence in dependence of number of prior treatment lines **(D)**, prior bendamustine treatment **(E)** or bone marrow infiltration **(F)**.

Abbreviations: CH, clonal hematopoiesis; VAF, variant allele frequency

## Figure S2

Presence of clonal hematopoiesis (CH) and association to collected cell yields. Boxplot displaying WBC **(A)**, ALC **(B)** and CD3+ cell count **(C)** before apheresis, and yields of CD3^+^ **(D)**, CD3^+^CD4^+^ **(E)** and CD3^+^CD8^+^ **(F)** cells in apheresis collections according to CH status.

Abbreviations: WBC, white blood cell count; ALC, absolute lymphocyte count

## Figure S3

Boxplot with overlaid individual data points comparing variant allele frequency (VAF) distribution of CH mutations according to manufacturing results.

Abbreviations: OOS, out-of-specification

## Figure S4

Prevalence of CH mutations ≥ 2% VAF in this cohort and associations with clinical factors. **(A)** Barplot showing prevalence of CH mutations ≥ 2% VAF according to patient age group. **(B)** Histogram displaying distribution of mutation count per patient. **(C, D, E)** Barplot showing CH ≥ 2% VAF prevalence in dependence of number of prior treatment lines **(C)**, previous bendamustine treatment **(D)** or bone marrow infiltration **(E)**.

Abbreviations: CH, clonal hematopoiesis; VAF, variant allele frequency

## Figure S5

Presence of CH mutations ≥ 2% VAF and associations with manufacturing results. (A) Boxplot with overlaid data points comparing variant allele frequency distribution of CH mutations ≥ 2% VAF according to manufacturing results. (B) Histogram comparing mutation count per patient between manufacturing result groups. (C) Barplot comparing relative mutation prevalence across genes by manufacturing groups.

Abbreviations: OOS, out-of-specification; CH, clonal hematopoiesis; VAF, variant allele frequency

## Figure S6

DNA damage response (DDR) mutations. Barplot displaying prevalence of mutations in DDR genes *TP53* and *PPM1D* according to manufacturing results group **(A)**, number of prior treatment lines **(B)** and previous bendamustine treatment **(C)**.

Abbreviations: OOS, out-of-specification

## Figure S7

Difference in CD3+ cell yields in apheresis collections by manufacturing outcome. P-values shown were adjusted for multiple testing.

Abbreviations: OOS, out-of-specification; CH, clonal hematopoiesis; VAF, variant allele frequency
